# Evaluation of Dietary Intake and Anthropometric Status in 1–9-Year-Old Children Living in Serbia: National Food Consumption Survey according to the EU Menu Methodology

**DOI:** 10.3390/nu14153091

**Published:** 2022-07-27

**Authors:** Milica Zeković, Jelena Milešević, Marija Takić, Marija Knez, Ivana Šarac, Agnes Kadvan, Mirjana Gurinović, Marija Glibetić

**Affiliations:** 1 Centre of Research Excellence in Nutrition and Metabolism, Institute for Medical Research, National Institute of Republic of Serbia, University of Belgrade, 11000 Belgrade, Serbia; jelena.milesevic@gmail.com (J.M.); marijapo2001@gmail.com (M.T.); marijaknez186@gmail.com (M.K.); ivanasarac@yahoo.com (I.Š.); mirjana.gurinovic@gmail.com (M.G.); mglibetic@gmail.com (M.G.); 2 Capacity Development Network in Nutrition in Central and Eastern Europe (CAPNUTRA), 11000 Belgrade, Serbia; k_agi@yahoo.com

**Keywords:** dietary intake assessment, anthropometric status, food consumption, children nutrition

## Abstract

The Serbian Food Consumption Survey among 1–9-year-old-children was conceptualized and conducted in compliance with the principles, established protocols, and guidelines of the EU Menu project between 2017 and 2021. Valid data were collected for 576 individuals (290 1–3-year-old toddlers and 276 3–9-year-old children). Regardless of age and gender category, the majority (68.80%) of children had normal weights according to the Body Mass Index-for-age classification system. The median daily energy intake was 1406.71 kcal with no differences between the settlement types. The overall median contributions of carbohydrates, protein, and fat to the total energy intake were 47.54%, 14.06%, and 37.88%, respectively. The proportions of the macronutrient intake deviated from the dietary reference values with compliance to the recommendations being particularly poor for fat and fiber. The consumption of energy-dense food groups such as meat and meat products, fat and oil, sugar, and confections was more pronounced among older children. The survey results provide a valuable insight into the nutritional status and dietary habits of toddlers and children 1–9 years old living in Serbia. They may serve as an evidence platform for public health programs, a valuable asset for decision-makers, and a reliable reference to guide nutritional policies, diet monitoring, and interventions targeting this population group in the future.

## 1. Introduction

A nutritionally appropriate, diversified, balanced, and safe diet is of paramount importance for health promotion and preservation over the course of one’s life [1]. A plethora of factors including socioeconomic status, cultural and environmental circumstances, food availability and affordability, knowledge, attitudes, and personal preferences interact in a dynamic and complex manner forming an individual’s time-specific dietary pattern. In childhood, optimal nutrition promotes intensive growth and fosters proper physical and psychological development, cognitive operability, and academic performance. Accordingly, food choices, nutrient intake, and dietary behavior in this delicate period may have a profound impact on shaping nutrition-related health trajectories [2].

The detrimental effects of early-life malnutrition are acknowledged from both short-term and long-term perspectives. The consequences of an inadequate diet during childhood include stunting, wasting, overweight, developmental delays, increased susceptibility to infections, and an intensified overall risk of adverse health outcomes [3]. Furthermore, these early-life disadvantages may interact with and aggravate lifestyle and nutrition factors later in life, leading to prolonged and persistent repercussions with particular emphasis on non-communicable diseases. Therefore, childhood is a period of critical vulnerability, but also an opportunity for establishing and consolidating healthy eating habits [4]. 

National food consumption surveys serve as an indispensable evidence platform for informing relevant policy decisions. They provide valuable population-level data for food intake, dietary patterns and nutrient adequacy evaluations, diet-related risk assessments, and the development of guidelines [5]. Furthermore, these surveys are significant instruments for malnutrition screening, the identification of disparities and critical areas of concern, and for the monitoring of applied interventions and their impact and effectiveness [6]. Within the European Food and Nutrition Action Plan, the WHO explicitly stimulates member-states to strengthen local surveillance programs and undertake nationwide nutrition surveys, thereby highlighting the need for valid, representative, and harmonized data [7]. Nevertheless, the provision of such surveys across Europe is rather inconsistent. A recent review reported that less than two-thirds of WHO European countries conducted national diet surveys since 1990, highlighting the Central and Eastern European region as the major data-gap area [6]. 

The Multiple Indicator Cluster Survey (MICS), carried out in Serbia in 2019 on a nationally representative sample and a sample of Roma households, provides high-quality, statistically robust, comparable, and internationally standardized data on the critical indicators of well-being among women, children, and adolescents [8]. The MICS program is an invaluable policy instrument and a source of credible information for the evaluation of the progress achieved towards nationally-defined objectives and global commitments, such as the Sustainable Development Goals (SDGs). Although addressing the nutritional status of children under five according to basic anthropometric indices, the duration of exclusive breastfeeding, and the prevalence of minimal dietary diversity, the scope and methodological frame of these surveys do not cover all-encompassing data regarding dietary patterns, food consumption, and nutrient intake among children. A Yugoslav study of atherosclerosis precursors in schoolchildren in Serbia, conducted from 1998 to 2003, was the first and until now remained the only country-level survey in Serbia based on comprehensive dietary assessment methods [9]. Therefore, there was an evident need for up-to-date reliable and robust dietary intake data and related information collected in accordance with internationally acknowledged, validated indicators and standardized methodologies [10]. 

Within the “What’s on the Menu in Europe?” (EU Menu) framework, the European Food Safety Authority (EFSA) has been leading a collective effort to establish a harmonized pan-European Food Consumption database featuring aligned methodological approaches, consistent information coding, and solid data-quality criteria with the potential of inter-country comparisons and analyses. The National Food Consumption Survey in Serbia, supported by the EFSA, was launched in 2017, with an aim to provide insight into children’s food consumption, nutrient adequacy, intake risks of potentially hazardous substances, and dietary trends [11]. The objective of the present study is the overview of the energy, macronutrient, and food group intake among one- to nine-year-old-children living in Serbia. 

## 2. Materials and Methods

### 2.1. Survey Design and Study Population

The Serbian Food Consumption Survey methodology details have been previously published [11]. In brief, the study was conceptualized and conducted in compliance with principles, established protocols, and guidelines of the EU Menu project between 2017 and 2021 [12,13]. The nationally representative sample comprised children residing in private households in the territory of the Republic of Serbia and was classified into two subgroups: (1) one- to three-year-old toddlers, and (2) three to nine year-old-children. A national population register updated with relevant population projections was used as the sampling frame. The recruitment was performed at the household level with age, sex, and residential region applied as three stratification layers. Only one subject was selected from each participating household and institutionalized individuals were considered ineligible. Study subjects were evenly distributed over four seasons and weekdays in order to capture the inter-seasonal variability in dietary patterns and day-to-day consumption fluctuations. The survey schedule covered the whole year including the festive calendar, i.e., national holidays and religious celebrations.

Informed written consent was obtained from all recruited subjects. The study was conducted in accordance with the Institutional Ethical board standards (Approval number: EO 123/2017, 8 December 2017, Institute for Medical Research, National Institute of the Republic of Serbia, University of Belgrade, Serbia) and the principles of the Helsinki declaration.

### 2.2. Data Collection 

Data collection was performed by trained interviewers with a project-specific national questionnaire compilation approved by EFSA. Acknowledging the relevance of family and social contextual factors in nutrition research, the general questionnaire collected data on sociodemographic variables for the parents/caretakers, as well as the general health and food-allergy status for children. Pre-defined answer options were arranged in compliance with the EU Menu methodology standards, classification systems, and nomenclature. Dietary intake assessment was based on twenty-four-hour food diaries reported for two non-consecutive days with at least one-week time distance to account for intraindividual food consumption variance. After each recording period, within debriefing face-to-face sessions, interviewers carefully reviewed the diaries with the parent/caretaker to clarify uncertainties, resolve possible omissions, probe for additional information or make necessary amendments, and thereby ensure adequate quality of the obtained data. The structural format of the diary was organized according to the stepwise procedure of the multiple-pass interview approach. All items were reported prospectively, i.e., at the point of consumption. A comprehensive set of collected information included meal type, place and time of each consumption occasion, food name and category (simple foods vs recipes), disaggregated ingredients in case of composite dishes, preparation procedures, estimated portion size, and if applicable, supplementary details regarding particular qualitative features such as fat and/or sugar content. Diverse approaches were applied to quantify food consumption depending on the available information and respondents’ preferences. In addition to natural units, standardized portions, and calibrated household measures, participants were encouraged to use the previously validated photographic Food Atlas specifically developed for the Balkan region [14]. Furthermore, precise mass (g) or volume (mL) were determined in case the exact data were presented on the label, or when the parent/caretaker measured the food quantity during the preparation or serving (e.g., infant formulae). 

### 2.3. Data Processing and Dietary Intake Assessment

Advanced software platform for nutrition research Diet Assess and Plan (DAP) was applied for data storage, processing, and dietary intake assessment. Encompassing electronic versions of standard food consumption and general questionnaires, portion size estimation picture books, Serbian food composition database (FCDB), and appropriate nutrient recommendation datasets, DAP was verified by the EFSA in the ring trial that took place within the initiative “Dietary monitoring tools for risk assessment” in 2014, and was used in numerous national, regional, and international projects [15,16,17]. The integrated FoodEx2 coding system enabled food matching between dietary questionnaires and the food composition database [18]. Nutrient estimates were based on the mean daily values calculated from the two nonconsecutive twenty-four-hour food diary replicates. Food group classification referred to categories proposed by EuroFIR and comprised milk/milk products, eggs/egg products, meat/meat products, seafood and related products, fat/oil, grains/grain products, nuts/seeds/kernel products, vegetable/vegetable products, fruit/fruit products, sugar/sugar products, beverages (non-milk), miscellaneous food products, and products for special nutritional use. Estimated energy intake was expressed in both kcal and MJ, protein intake in absolute values (g) and relative to body weight (g/kg body mass), and percentage contribution to total energy intake was calculated for all macronutrients and saturated, monounsaturated, and polyunsaturated fatty acids. Nutrient adequacy assessment was performed against both Serbian national [19] and EFSA [20] age-appropriate sets of recommendations as summarized in Table 1. 

### 2.4. Anthropometric Assessment

Prior to fieldwork, all the participating interviewers completed the training course for performing anthropometric measurements of the population of children and used identical equipment. Weight and length/height evaluation was performed during the personal interview and followed the WHO child growth assessment protocols. Portable stadiometers (Seca 213, Secagmbh & Co., Hamburg, Germany) with 0.1 cm accuracy were applied for height measurement. Weight was measured in light clothing using a digital balance with taring capability, calibrated to 0.1 kg (Tanita BC-545N, Tanita, Tokyo, Japan). Body mass index (BMI) was calculated as the ratio of weight (in kg) and recumbent length or standing height (in m). Subjects were classified based on nutrition conditions using the BMI-for-age standardized charts and z-score- (i.e., standard deviations (SD)) cut-off points proposed by the WHO [21]. The following BMI-for-age categories were determined: severely underweight—z_sd_ ≤ −3; underweight—−3 < z_sd_ ≤ −2; normal weight—−2 < z_sd_ ≤ 1; possible risk of overweight—1 < z_sd_ ≤ 2 for children below 60 months of age; overweight—2 < z_sd_ ≤ 3 and 1 < z_sd_ ≤ 2 for subjects bellow 60 months of age and older children, respectively; and finally, obese—z_sd_ > 3 and—z_sd_ > 2 for subjects bellow 60 months of age and 5 to 9-year-old children, respectively. For children under 60 months of age, weight-for-length/height indicator was applied as a complementary metric for overweight/obesity estimates in accordance with the WHO age and sex appropriate standards [21].

### 2.5. Statistical Analyses

Statistical data analysis was performed with IBM SPSS Statistics 22 (*SPSS Inc*. Chicago, IL, USA). In this study, measurable data were characterized by measures of variability (the mean and standard deviation), whereas attributive data were in absolute numbers and frequencies. The normality of the variable distribution was explored with the *Shapiro–Wilk* test. The differences in continuous data with normal distribution were assessed with the Student’s *t*-test; otherwise, in case of skewed distribution, the *Mann–Whitney* test was applied. For energy and relevant macronutrients, results are presented in terms of the mean and standard deviation as well as the distribution of the estimated intake per day (percentiles 5, 25, 50, 75, and 95). The *Kruskal–WallisH* test was applied to explore the differences in macronutrient and energy intake between the geographical areas. The inferential *Chi-square* test was used to explore the relationships between categorical variables. Statistical hypotheses were analyzed at the 0.05 significance level. 

## 3. Results

A total of 774 eligible subjects (403 girls and 371 boys) were approached during the recruitment process and parents or legal guardians of 580 of the prospective subjects voluntarily agreed to participate. Complying with the study protocol and EFSA guidelines, subjects with only one twenty-four-hour food diary available (*n* = 4) were disqualified and excluded from the analyses. Accordingly, valid data were collected for 576 individuals (290 1–3-year-old toddlers and 276 3–9-year-old children), yielding an overall response rate of 74.41%. No statistically significant difference in the response rate was observed regarding the age subgroup or gender classification. The regional distribution of subjects corresponded with the a priori defined strata and the national geographical population density statistics. Nevertheless, subjects residing in urban households were overrepresented compared to those living in rural areas (85.42% vs. 14.58%). The vast majority of children (98.26%) followed a conventional (omnivorous) diet. Based on the parental/caretaker reports, only one participant in each age sub-category practiced veganism/vegetarianism, while less than 2% had a diet adjusted to a specific health condition such as celiac disease or diabetes. The prevalence of confirmed food allergies ranged from 2.07% in the toddlers’ group to 5.59% among older children with an even gender-based distribution. The overview of the study sample characteristics including parental education, occupation, and professional profile is presented in Table 2. 

Based on anthropometric measurements, the median length/height and weight of the children were 101.0 cm, range: 53.0–155.0 cm (under 60 months of age: 92.0 cm, range: 53.0–128.0 cm and 5–9-year-old: 128.0 cm, range: 83.0–155.0 cm) and 16.0 kg, range: 8.0–51.0 kg (under 60 months of age: 14.0 kg; range: 8.0–30.0 kg, and 5–9-year-old: 25.0 kg, range: 14.0–51.0 kg), respectively. A sample nutritional status overview based on the BMI-for-age categories is displayed in Table 3. Regardless of age and gender category, the majority (68.80% on a total sample level) of children had normal weights according to the BMI-for-age classification system. A total of 63 subjects (10.94%) were characterized as overweight, 36 (6.25%) as obese, and for an additional 68 (11.80%) aged under 60 months the possible risk of being overweight was determined. Based on the weight-for-length/height indicator, 72 individuals under 60 months of age (19.1% of age subsample, 40 girls and 32 boys) were assigned in the overweight risk category. Furthermore, 29 individuals (16 girls and 13 boys) were overweight and 20 (6 girls and 15 boys) were obese, accounting for 7.9% and 5.3% of the age subsample, respectively. The proportion of subjects allocated in these categories corresponded well with the estimates based on the BMI-for-age classification system. Most misclassifications occurred near the percentile cut-off points, with no differences regarding sex and age subcategories. No association was observed between gender and overweight/obesity prevalence regardless of the metric applied. Concerning the sample’s geographical distribution, the proportion of overweight/obese subjects in Western Serbia and the Šumadija region (23.8%) was significantly higher than in other analyzed areas (*p* < 0.05). Furthermore, overweight and obese individuals were more prevalent in urban (26.8%) in comparison with rural households (15.7%) (*p* < 0.05). Nevertheless, the proportion of underweight children was also significantly higher in urban compared to rural areas (15.7% vs. 1.2%, *p* < 0.05).

The distribution of estimated energy and macronutrient intake (including mean values and percentiles) across age and gender groups is presented in Table 4. Median daily energy intake was 1406.71 kcal, range: 495.27–3156.36 kcal with no differences between settlement types. The participants residing in the South-Eastern Serbia region had a significantly higher energy intake compared to other geographical strata (χ(3) = 21.892, *p* < 0.001) (Table 5). Although not reaching the statistical significance threshold neither at the total sample level nor within the age-subgroup analyses, boys had a higher energy intake than girls (1513.51 ± 474.08 kcal vs.1473.86 ± 462.27 kcal). No gender-wise differences were observed concerning the estimated intake of total carbohydrates, fat, and protein. Nevertheless, the intake of protein relative to body weight was significantly higher among female subjects aged 1–3 years compared to their male peers (3.67 ± 1.13 g/kg body weight vs. 3.38 ± 1.13 g/kg body weight, *p* < 0.05).Among toddlers, expectedly, the absolute intake of energy, carbohydrates, protein, fat, and fiber increased with age (r = 0.401, r = 0.334, r = 0.393, and r = 0.278, all *p* < 0.001). Analogously, in 3–9-year-old children, a positive correlation was recorded between the absolute intake of energy, carbohydrates, protein, fat, and fiber and age (r = 0.326, r = 0.243, r = 0.281, r = 0.342 and r = 0.128, all *p* < 0.001). Nevertheless, in this group, the protein intake relative to body weight decreased with age (r= −0.392, *p* < 0.01). The percentage contribution of macronutrients to total energy remained consistent across the subgroup age span except for fat (1–3 year-old toddlers = 0.153, *p* < 0.01; 3–9 year-old children: r = 0.186, *p* < 0.01). The overall median contributions of carbohydrates, protein, and fat to total energy intake were 47.54%, 14.06%, and 37.88%, respectively. A total of 356 (61.81%) subjects had 45–60% of their energy derived from their intake of carbohydrates, while for 30.00% of toddlers and 38.11% of 3–9-year-old children the respective contribution was below the lower reference intake limit of 45% with an even distribution among girls (35.35%) and boys (34.78%). The median contribution of fat to total energy intake among toddlers was 36.68%, with 24.13% and 32.07% of participants falling into the age-appropriate national and EFSA reference intake ranges, respectively. The fat intake was even higher in the older age sub-group with more than two-thirds (76.20%) of subjects exceeding the 35% of energy derived from fat. The contribution of carbohydrates and monounsaturated fatty acids was significantly higher in Vojvodina (northern autonomous region of Serbia) in comparison with other geographical areas (χ(3) = 12.246, *p* < 0.01, χ(3) = 11.905, *p* < 0.01, respectively) (Table 5). The protein intake relative to body weight was above the age-specific average requirements defined by the EFSA in all the participants, with estimated values almost triply exceeding the population reference intake thresholds. The median dietary fiber intake per day among toddlers was 10.68 g (range: 1.11–38.56 g), surpassing the age-specific adequate intake (AI) level of 10 g/day, as proposed by the EFSA. A total of 171 individuals (58.66%) in this age group reached that threshold with no differences between the female and male participants. The median estimated intake of fiber among 4–6-year-olds (13.00 g, range: 4.32–29.23 g) was below the AI level for that age subgroup (i.e., 14 g/day) with a slightly higher proportion of boys achieving the reference cut-off point (*n* = 32, 43.24%) compared to their female peers (*n* = 27, 40.90%). Similar observations were made in the oldest subgroup, i.e., among those7–9 years old: although showing an age-related increasing trend, the median fiber intake (13.83 g, range: 5.77–44.71 g) remained below the AI level, with only 36.73% (girls: 34.25%and boys: 39.73%) of participants achieving the 16 g/day target. Children living in rural households consumed more dietary fiber daily than those residing in urban areas (12.31 g, range: 1.11–44.71 g vs. 11.09, range: 2.20–28.58 g, respectively, *p* < 0.05). Moreover, the intake of dietary fiber was significantly higher in the South-Eastern Serbia region than in other analyzed areas (χ(3) = 29.372, *p* < 0.001).

The contribution of the food groups to the total energy intake, energy intake from carbohydrates, protein, fat, dietary fiber, and saturated fatty acids across age and gender categories are summarized in Table 6. Based on the dietary records, grains and grain products were the most commonly consumed food group (eaten by almost 100% of the study participants), followed by milk and milk products (96.87%), vegetables and vegetable products (95.31%), and fruits and fruit products (93.58%).The share of the consumers of meat and meat products among the participants were fourfold higher compared to the consumers of seafood and related products (84.90% vs. 20.31%). Sugar and related products occurred in dietary records of approximately two thirds of respondents, while less than 10% consumed nuts, seeds, and kernel products. Significant gender-wise differences in the food group consumption patterns were not observed in the analyzed sample (data not shown). Overall, grains and grain products accounted for more than one-third of the total energy intake. Furthermore, these food items were the dominant source of energy intake for carbohydrates and dietary fiber. In addition to grains, fruit, vegetables, confections, and associated products were the dominant sources of carbohydrates. Unsurprisingly, energy and nutrient-dense animal source food groups, i.e., milk, meat, and their related products were significant contributors to the protein and fat intake. Moreover, the percentage of energy intake from saturated fatty acids was most apparent in the milk and milk product category. The food group consumption patterns fluctuated between the two age sub-groups. The consumption of milk and milk products, fruit and fruit products, and products for special nutritional use (formulas) was more pronounced among toddlers. In contrast, 3–9-year-oldchildren had a greater energy intake derived from meat and meat products, fat and oil, sugar and sugar products, and miscellaneous foods.

## 4. Discussion

The Serbian Food Consumption Survey, conducted on a nationally representative sample (*n* = 576) in line with the rigorous standards and methodological framework of the EU Menu project established by the EFSA, provides much-needed insight into the nutritional status and dietary habits of toddlers and children 1–9 years old living in Serbia. 

An individual’s overall health is influenced by genetic and epigenetic legacies; intrauterine stressors; environmental exposures; behavioral factors; interpersonal relationships; sociopolitical, cultural, and economic structures; norms; and opportunities. Acknowledging the importance of proper childhood development in the pursuit of health across individuals’ lifespan, the World Health Organization (WHO) advocates for the adoption and implementation of a life-course approach accentuating the need for “early, timely, appropriate and collective” actions [22]. Practical, sustainable, and evidence-based strategies and policies targeting the optimization of nutrition among children could make a major contribution to mitigating the humanistic, clinical, and financial impact of acute complications and late sequelae of young-age malnutrition. A multisectoral food system approach systematically and comprehensively engaging various stakeholders with coordinated, coherent, and complementary actions across governmental and social structure levels is the most effective manner for addressing the complexity of nutritional issues while concomitantly ensuring safe, affordable, and sustainable diets [23,24,25].

Approximately two-thirds of the study participants had a normal weight according to the WHO BMI-for-age classification system. Nevertheless, 10.94% of children were characterized as overweight, 6.25% as obese, and for an additional 11.80% of participants under 5 years of age the anthropometric measurements suggested a possible risk of becoming overweight. These results are in accordance with the data reported in the Multiple Indicator Cluster Survey findings conducted in Serbia in 2019 [26]. Furthermore, the findings of our study corresponded well with the UNICEF/WHO/World Bank Group Joint Child Malnutrition Estimates of the prevalence of overweight among children in Serbia. With the modeled estimate value of 10.8%, Serbia was allocated in the high overweight prevalence category based on the country-level threshold classification system proposed by the WHO-UNICEF Technical Advisory Group on Nutrition Monitoring [27]. Excess body weight is a complex disorder with a rising prevalence worldwide, implying complex and interconnected genetic, metabolic, psychological, socioeconomic, and environmental etiopathogenetic factors [28]. Paradoxically concomitant with undernutrition, the prevalence rates of overweight and obesity in childhood have reached alarming levels, especially in developed and developing countries [29]. Furthermore, with respect to obesity during childhood, and in particular during adolescence, there is a high risk for the persistence of the condition into adulthood [30]. Obesity at a young age has been associated with an increased morbidity burden and numerous medical and socio-emotional repercussions. These conditions include, but are not limited to: cardiovascular diseases, metabolic disturbances, diabetes mellitus, sleep apnea, hepatic steatosis, musculoskeletal issues, dermatologic problems, menstrual abnormalities, asthma, depression, poor socialization, and anxiety [31]. Given the multifaceted nature of both the causes and consequences of childhood obesity, the prevention and management of this problem require actions across a variety of contexts such as family, school, community, the healthcare system, and governmental public health services [32]. The identification of children with excess body weight and the application of age-appropriate, culturally sensitive evidence-based measures and interventions may lower the obesity rates as well as the public health and societal burden of obesity in adulthood [33,34,35,36]. 

Analyses of the urban-rural disparities in nutritional status revealed that the deviation from normal body weight towards both ends of the spectrum was more prevalent in urban settings compared to rural households. This may be attributed to a westernized dietary pattern, less physical activity, increased exposure to highly processed food with poor nutritional quality, distancing from the traditional food consumption model, and meal plan irregularities [37]. Worldwide data suggest that compared to rural circumstances, urban dietary trends incline towards energy-dense high-fat food; an increased consumption of refined, more polished, and milled grains; and a higher intake of animal-source products, sugar, salt, and (ultra)processed food [38]. Furthermore, there is an additional impact of intensified susceptibility and responsiveness to the mass media and marketing activities of the commercial food sector and an increased consumption of food prepared outside of the home. The “nutrition transition” phenomenon encompasses concurrent changes in dietary and energy expenditure patterns, coinciding with economic development, urbanization, demographic shifts, modernization, epidemiological factors, food system changes, and technological advancements [8]. Early-life exposure to such practices is of particular concern since it may aggravate the adverse health effects of an unfavorable energy balance and diet quality [39]. An improved comprehension of the drivers and outcomes of the discrepancies between the eating habits, lifestyle, nutritional status, and body composition among urban and rural residents may contribute to an enhanced assessment of their health demands and the development of interventions tailored according to their respective settings [40].

The mean estimated energy daily intake of 6.25 ± 1.98 MJ was comparable with reports from other European countries, and expectedly, higher values were recorded for boys and older children. The consumption of energy-dense food groups such as meat and meat products, fat and oil, sugar, and confections was more pronounced among older children. These trends may be related to physiological factors, increased requirements due to developmental and physical activity factors, diet diversification, the broadening of food repertoires, and the increased eating autonomy that develops with aging [41,42]. There were no intake discrepancies regarding particular food groups between girls and boys, suggesting that gender-associated food–preference differences may occur later in life [40]. The nutritional composition analysis revealed certain deviations regarding the proportions of the macronutrient intake and dietary reference values. Congruently with the comprehensive overview of the nationally representative dietary surveys encompassing 53 countries in the WHO’s Europe remit, the compliance with the recommendations was particularly poor for fat and fiber intake [41]. With the overall median estimation of 37.88% for the energy derived from fat, more than two-thirds of the study participants exceeded the 35% threshold. Fat and energy intake are closely correlated, which makes it perplexing to isolate their individual effects on bodyweight measures. Although high-quality long-term trials and properly designed prospective studies are warranted to fully elucidate the health effects of a high-fat diet in the population of children, there is a substantial body of evidence associating such eating patterns with cardiometabolic risk factors and adiposity indices in children [43]. Along with other complimentary measures, conducted at the individual, community, and population levels, an adjustment of fat consumption may contribute to the maintenance of regulated energy balance for a healthy weight and the prevention of overweight and obesity in youth [3]. 

Although showing an age-related increasing trend in terms of absolute daily intake values, the proportion of subjects reaching the dietary fiber AI threshold decreased from toddlers towards older subgroups in the analyzed children cohort. Only in the toddlers’ group did the median estimated intake per day surpass the age-specific AI level. In both succeeding subgroups, namely the 4–6- and 7–9-year-old participants, more than 60% remained below the AI value, with a slightly higher proportion of boys reaching the reference cut-off point than their female counterparts. The observed trends might be partially attributed to differences in the organizational framework and practical perspectives for collective child nutrition in Serbia. There are official, legally endorsed recommendations for coordinating, monitoring, and implementing the collective feeding programs for children in kindergartens and primary schools [19,44]. In these institutions, meal planning is performed by certified professionals in accordance with the age-appropriate nutritional normative standards. Although not including specific values for dietary fiber, the recommendations for the representation of food groups in daily/weekly menus as well as the distribution of energy and macronutrients across the meals adjusted for the duration of the child’s stay are laid down in official guidelines for both institution levels. The kindergarten program encompasses three mandatory meals (i.e., breakfast, snack, and lunch), and one additional discretionary snack, presumably providing 75%of the total daily energy needs. However, in primary schools, there is wide variability regarding the meal-offer coverage, which depends on the operational, logistical, and organizational resources. Furthermore, while kindergarten feeding programs include all the enrolled children, organized nutrition in primary schools is optional, and the decentralized nature of school-meal programs in Serbia hinders the availability of precise data regarding the proportion of students who benefit from school feeding. Dietary fiber, as an essential nutrient, confers a plethora of both short-term and long-term functional and physiological health benefits. The most common effect associated with a sufficient fiber intake is the improvement of the digestive process and overall gastrointestinal health, including laxation. Nonetheless, fiber benefits extend beyond the gut function, and include cholesterol reduction, glycemic control, the prevention of cancer and cardiovascular diseases, and weight management, as well as supporting the immune system and proper cognition in children [45,46,47]. The majority of official guidelines propose recommendations only in terms of quantity, neglecting to address the relevant physico-chemical properties, fermentability, bulking, and dietary sources. However, the diversity of fiber types in food, the heterogeneity of these types’ physiological effects, and the complexity of their functional properties provide the scientific rationale for a consideration of the qualitative features in addition to the simple total daily consumption amount [48]. The data retrieved from numerous national dietary surveys suggest that children in most industrialized countries in Europe, North America, and Oceania fail to achieve the recommended intake levels [49,50]. Given the potential adverse effects of a low-fiber intake on pediatric diets, parents/caretakers should be counseled to encourage children to consume a diverse diet including more whole-grain products, fruits, vegetables, legumes, nuts, seeds, and other fiber-rich food sources. Furthermore, efforts should be made to provide general public health guidance on dietary fiber sources, recommendations, and the importance of health maintenance and disease prevention. 

The physical and social environment are significant determinants of the eating patterns in the pediatric population. Parents’ nutritional behaviors and attitudes, as well as peers’ dietary habits, shape children’s food appeal and feeding style. The availability and accessibility of and repeated exposure to healthy foods are crucial to developing familiarity and acceptance [51]. It is challenging to assess the exact adequacy of the dietary intake of the food groups in the analyzed cohort as country-specific Food-Based Dietary Guidelines (FBDG) for the Serbian population do not exist at this stage [52]. The development of such a tool would help to better understand the quality of the diets of Serbian children and toddlers, identify potential nutritional challenges in this vulnerable population, and enable the timely addressalof specific inconsistencies. The WHO, FAO, and EFSA encourages member states do develop national FBDG using a multi-disciplinary stepwise approach with a particular focus on the diet–disease relationships relevant to the population of interest [53]. Well-designed, carefully structured dietary guidelines that take into account generally established nutritional principles, environmental impact, and current scientific evidence are a prerequisite for the development of policies aimed at shifting the consumption patterns of children towards healthier directions, thereby ensuring long-term beneficial effects and the prevention of diet-related diseases later in life [54,55,56].

Certain limitations of the present study should be acknowledged. The cross-sectional design prevents us from drawing any causal inferences, and further longitudinal research is warranted to acquire a better understanding of the potential nutritional challenges and health determinants among children living in Serbia. The intricacy of the study protocol, the high degree of parental/caretaker involvement required, the lack of financial participation incentives, and the obsolescence of the Census data were major concerns that hindered recruitment. Despite the effort invested in the nationally representative sampling, given the voluntary nature of participation in this study, selection bias to some extent cannot be completely precluded, thus limiting the generalizability of the presented findings. Among the key issues in nutritional epidemiology are measurement error and the inherent limitations of self and proxy-reported dietary consumption data. Nevertheless, the data collection was conducted by trained professionals with expertise in nutrition research in accordance with internationally recognized methodological guidance in a prospective manner, taking into account seasonal, weekly, and intraindividual dietary variability. Furthermore, comprehensive quality assurance procedures were employed across the study’s critical points including sampling/recruitment, fieldwork management, and data cleaning.

## 5. Conclusions

Given the scarcity of previously available data, the presented Serbian Food Consumption Survey may serve as a fundamental evidence platform for public health programs, a valuable asset for decisionmakers, and a reliable reference to guide nutritional policies, diet monitoring, and interventions targeting the population of toddlers and children in the future. Furthermore, the application of a harmonized methodological approach established under the EU Menu project facilitates comparative analyses across the continent and contributes to a common pan-European food consumption database.

## Figures and Tables

**Table 1:** Comparative overview of EFSA and Serbian daily requirements for carbohydrates, protein, and fat for 1–9-year-old children.

EFSA			RS		
Total carbohydrates (g/day)					
Age		RI		RDI	RI
1–17 years		45–60 TE%	1–3 years	150–180 g	50–60 TE%
			3–7 years	200–240 g	50–60 TE%
Total fat (g/day)					
1 year		35–40 TE%	1–3 years	40–47 g	30–35 TE%
2–3 years		35–40 TE%	3–7 years	53–62 g	
4–17 years		20–35 TE%			
Age	AI				
Dietary fiber (g/day)					
1–3 years	10				
4–6 years	14				
7–10 years	16				
Protein (g/kg bw per day)					
Age	AR	PRI		RDI	
12–17 months	0.95	1.14	1–2 years	1.20	
18–23 months	0.85	1.03			
2 years	0.79	0.97	2–3 years	1.15	
3 years	0.73	0.90			
4 years	0.69	0.86	3–5 years	1.10	
5 years	0.69	0.85			
6 years	0.72	0.89	6–7 years	1.00	
7 years	0.74	0.91			
8 years	0.75	0.92			
9 years	0.75	0.92			

RS—Serbian national age-appropriate set of recommendations; EFSA—European Food Safety Authority dietary reference values; TE—total energy intake; AR average requirements; AI—adequate intake; RDI—recommended daily intake; PRI—population reference intake.

**Table 2:** Characteristics of the study population.

Variable	Toddlers *n* = 290	Children *n* = 286	Total Sample *n* = 576
Gender, *n* (%)			
Female	141 (48.62%)	139 (48.60%)	280 (48.61%)
Male	149 (51.38%)	147 (51.40%)	296 (51.39%)
Age in years, $\bar{x}$ ± SD	1.98 ± 0.64	6.01 ± 1.87	3.98 ± 2.43
Response rate, *n* (%)	73.98%	74.87%	74.42%
Distribution per geographical region, *n* (%)			
Belgrade (capital city) region	72 (24.93%)	64 (22.38%)	136 (23.61%)
Vojvodina region	78 (26.90%)	78 (27.27%)	156 (27.08%)
Region of Šumadija and Western Serbia	84 (28.97%)	84 (29.37%)	168 (29.17%)
South-Eastern Serbia region	56 (19.31%)	60 (20.98%)	116 (20.14%)
Dietary pattern of a child, *n* (%)			
Conventional	287 (98.97%)	279 (97.55%)	566 (98.26%)
Vegan/vegetarian	1 (0.34%)	1 (0.35%)	2 (0.35%)
Diet related to health conditions (e.g., celiac disease, diabetes)	2 (0.69%)	6 (2.10%)	8 (1.39%)
Prevalence of confirmed food allergies, *n* (%)	6 (2.07%)	16 (5.59%)	21 (3.65%)
Household size and composition, $\bar{x}$ ± SD			
People per household	3.79 ± 0.97	4.08 ± 1.06	3.94 ± 1.03
Household members ≥ 18 years old	2.19 ± 0.71	2.33 ± 0.84	2.26 ± 0.78
Household members 10–17 years old	0.16 ± 0.42	0.43 ± 0.62	0.30 ± 0.55
Household members < 10 years	1.51 ± 0.62	1.49 ± 0.61	1.50 ± 0.62
Child lives with, *n* (%)			
Both parents	272 (93.80%)	263 (91.96%)	535 (92.88%)
Only mother	17 (5.86%)	20 (6.99%)	37 (6.42%)
Only father	0 (0.00%)	1 (0.35%)	1 (0.17%)
Other	1 (0.34%)	2 (0.70%)	3 (0.52%)
Highest level of formal education- mother; father, *n* (%)			
ISCED 0: less than primary education attained	0 (0.00%); 5 (1.72%)	2 (0.70%); 4 (1.40%)	2 (0.34%); 9 (1.56%)
ISCED 1: Primary education	1 (0.34%); 3 (1.03%)	1 (0.35%); 7 (2.45%)	2 (0.34%); 10 (1.74%)
ISCED 2: Lower secondary education	11 (3.79%); 9 (3.10%)	14 (4.90%); 14 (4.90%)	25 (4.34%); 23 (3.99%)
ISCED 3: Upper secondary education	78 (26.9%); 100 (34.48%)	94 (32.87%); 112 (39.16%)	172 (29.86%); 212 (36.81%)
ISCED 4/5: Post-secondary/Short-cycle tertiary education	25 (8.62%); 14 (4.83%)	23 (8.04%); 30 (10.49%)	48 (8.33%); 44 (7.64%)
ISCED 6: Bachelor’s or equivalent level	109 (37.59%); 107 (36.90%)	101 (35.31%); 77 (26.92%)	210 (36.46%); 184 (31.94%)
ISCED 7/8: Master’s/Doctoral or equivalent level	66 (22.76%); 52 (17.93%)	51 (17.83%); 41 (14.33%)	117 (20.31%); 93 (16.15%)
Employment status—mother; father, *n*(%)			
Unemployed	42 (14.48%); 8 (2.76%)	39 (13.64%); 16 (5.59%)	81 (14.06%); 24 (4.17%)
Working for pay or profit	193 (66.55%); 268 (92.41%)	217 (75.87%); 252 (88.11%)	410 (71.18%); 520 (90.28%)
Pupil, student, further training, unpaid work experience	4 (1.38%); 1 (0.34%)	1 (0.35%), 0 (0.00%)	5 (0.87%); 1 (0.17%)
In retirement or early retirement or has given up business	0 (0.00%); 0 (0.00%)	0 (0.00%), 2 (0.70%)	0 (0.00%); 2 (0.35%)
Maternity, parental, or sick leave	38 (13.10%); 0 (0.00%)	17 (5.94%); 0 (0.00 %)	55 (9.55%); 0 (0.00%)
Permanently disabled	0 (0.00%); 0 (0.00%)	0 (0.00%), 1 (0.35%)	0 (0.00%); 1 (0.17%)
In compulsory military or community service	0 (0.00%); 1 (0.34%)	1 (0.35%), 1 (0.35%)	1 (0.17%); 2 (0.35%)
Fulfilling domestic tasks	10 (3.45%); 0 (0.00%)	7 (2.45%), 1 (0.35%)	17 (2.95%); 1 (0.17%)
Not applicable/Other	3 (1.03%); 12 (4.14%)	4 (1.40%); 13 (4.55%)	7 (1.22%); 25 (4.34%)
Professional profile—mother; father, *n* (%)			
Managers	19 (6.55%); 39 (13.45%)	20 (6.99%); 41 (14.34%)	39 (6.77%); 80 (13.88%)
Professionals	92 (31.72%); 71 (24.48%)	72 (25.17%); 47 (16.43%)	164 (28.47%); 118 (20.49%)
Technicians and associate professionals	29 (10.00%); 39 (13.45%)	25 (8.74%); 43 (15.03%)	54 (9.38%); 82 (14.24%)
Clerical support workers	45 (15.51%); 11 (3.79%)	50 (17.48%); 14 (4.90%)	95 (16.49%); 25 (4.34%)
Service and sales workers	26 (8.97%); 31 (10.69%)	32 (11.19%); 38 (13.29%)	58 (10.07%); 69 (11.98%)
Skilled agricultural, forestry, and fishery workers	1 (0.34%); 5 (1.72%)	0 (0.00%); 1 (0.35%)	1 (0.17%); 6 (1.04%)
Craft and related trades workers	11 (3.79%); 8 (2.76%)	12 (4.20%); 21 (7.43%)	23 (3.99%); 29 (5.03%)
Plant and machine operators, and assemblers	2 (0.69%); 22 (7.59%)	0 (0.00%), 13 (4.55%)	2 (0.35%); 35 (6.08%)
Elementary occupations	8 (2.76%); 6 (2.07%)	14 (4.90%); 11 (3.85%)	22 (3.82%); 17 (2.95%)
Armed forces occupations	3 (1.03%); 6 (2.07%)	1 (0.35%); 3 (1.05%)	4 (0.69%); 9 (1.56%)
Other	54 (18.62%); 52 (17.93%)	60 (20.98%); 54 (18.88%)	114 (19.79%); 106 (18.40%)
Settlement type, *n* (%)			
Urban	249 (85.86%)	243 (84.97%)	492 (85.42%)
Rural	41 (14.14%)	43 (15.03%)	84 (14.58%)

**Table 3:** Nutritional status overview based on Body mass index (BMI)-for-age categories for a nationally representative sample of children aged 1–9 years living in Serbia (*n* = 576).

Sample Group (Age Categories)	BMI-for-Age Categories *					
	Severely Underweight	Underweight	Normal Weight	Possible Risk of Overweight	Overweight	Obese
Children <60 moths (*n* = 376)	8 (2.1%)	15 (4.5%)	230 (61.2%)	68 (18.1%)	32 (8.5%)	23 (6.1%)
Girls (*n* =176)	3 (1.7%)	8 (4.5%)	104 (59.1%)	38 (21.6%)	15 (8.5%)	8 (4.5%)
Boys (*n* =200)	5 (2.5%)	7 (3.5%)	126 (63.0%)	30 (15.0%)	17 (8.5%)	15 (7.5%)
Children 5–9 years (*n* = 200)	3 (1.5%)	4 (2.0%)	149 (74.5%)	NA	31 (15.5%)	13 (6.5%)
Girls (*n* =176)	0 (0.0%)	2 (1.9%)	83 (79.8%)	NA	16 (15.4%)	3 (2.9%)
Boys (*n* =200)	3 (2.1%)	3 (3.1%)	66 (66.8%)	NA	15 (15.6%)	10 (10.4%)

* severely underweight: z_sd_ ≤ −3; underweight: −3 < z_sd_ ≤ −2; normal weight: −2 < z_sd_ ≤ 1; possible risk of overweight: 1 < z_sd_ ≤ 2 for children below 60 months of age; overweight: 2 < z_sd_ ≤ 3 and 1 < z_sd_ ≤ 2 for subjects bellow 60 months of age and 5–9 years old children, respectively; obese: z_sd_ > 3 and: z_sd_ > 2 for subjects bellow 60 months of age and 5–9 years old children, respectively; NA—not applicable.

**Table 4:** Distribution of daily energy and macronutrients intake across age and gender categories in a nationally representative sample of children 1–9 years of age living in Serbia (*n* = 576).

Variable	Girls	Boys	Girls and Boys					
	$\bar{x}\;\pm \;\mathrm{SD}$	$\bar{x}\;\pm \;\mathrm{SD}$	$\bar{x}\;\pm \;\mathrm{SD}$	P5	P25	P50	P75	P95
Energy (kcal)								
toddlers	1267.44 ± 361.01	1299.53 ± 395.17	1283.93 ± 378.63	733.69	1038.14	1237.71	1456.06	2005.46
children	1683.24 ± 460.58	1730.41 ± 470.20	1707.48 ± 465.33	1016.57	1377.17	1652.01	1991.16	2600.88
total sample	1473.86 ± 462.27	1513.51 ± 474.08	1494.24 ± 473.61	865.72	1176.07	1406.71	1739.80	2341.23
Energy (MJ)								
toddlers	5.30 ± 1.51	5.43 ± 1.65	5.37 ± 1.58	3.07	4.34	5.18	6.09	8.39
children	7.04 ± 1.93	7.24 ± 1.97	7.14 ± 1.95	4.25	5.76	6.91	8.33	10.88
total sample	6.17 ± 1.93	6.33 ± 2.03	6.25 ± 1.98	3.62	4.92	5.89	7.28	9.80
Carbohydrates (g)								
toddlers	149.97 ± 45.91	158.15 ± 48.48	154.17 ± 47.34	84.76	124.65	146.61	179.14	233.30
children	193.42 ± 56.05	201.01 ± 56.53	197.32 ± 56.33	116.98	159.83	191.86	227.00	302.74
total sample	171.54 ± 55.54	179.44 ± 56.76	175.60 ± 56.26	102.08	137.41	167.87	204.66	286.31
Carbohydrates (%TE)								
toddlers	47.62 ± 7.79	49.01 ± 7.07	48.33 ± 7.44	36.64	43.85	48.37	52.83	60.45
children	46.13 ± 5.97	46.91 ± 6.91	46.53 ± 6.47	35.95	42.64	46.73	50.45	56.84
total sample	46.88 ± 6.97	47.97 ± 7.06	47.44 ± 7.03	36.14	43.27	47.54	51.57	59.11
Protein (g)								
toddlers	46.26 ± 13.51	46.20 ± 15.72	46.23 ± 14.67	25.68	36.72	44.13	53.91	73.96
children	60.51 ± 17.55	61.28 ± 19.31	60.90 ± 18.45	34.89	48.47	58.72	71.83	91.88
total sample	53.33 ± 17.17	53.69 ± 19.12	53.52 ± 18.18	29.61	40.58	51.08	63.99	86.46
Protein (g/kg body mass)								
toddlers	3.67 ± 1.13	3.38 ± 1.13	3.52 ± 1.14	1.91	2.76	3.40	4.13	5.52
children	2.66 ± 0.90	2.69 ± 0.93	2.68 ± 0.91	1.42	2.03	2.56	3.22	4.27
total sample	3.17 ± 1.14	3.04 ± 1.09	3.10 ± 1.11	1.54	2.32	2.97	3.69	5.08
Protein (%TE)								
toddlers	14.76 ± 2.49	14.22 ± 2.47	14.49 ± 2.49	10.91	12.83	14.06	15.99	18.96
children	14.50 ± 2.35	14.16 ± 2.32	14.33 ± 2.34	10.94	12.83	14.06	15.62	18.17
total sample	14.64 ± 2.42	14.19 ± 2.40	14.41 ± 2.42	10.87	12.82	14.06	15.84	18.63
Fat (g)								
toddlers	53.61 ± 21.40	53.57 ± 20.35	53.59 ± 20.84	25.60	40.35	50.00	62.70	91.00
children	74.17 ± 24.67	75.69 ± 25.53	74.95 ± 25.08	40.45	57.24	72.52	90.26	121.91
total sample	63.82 ± 25.24	64.56 ± 25.56	64.20 ± 25.38	30.68	45.35	60.05	76.43	112.34
Fat (%TE)								
toddlers	37.62 ± 7.44	36.76 ± 6.53	37.18 ± 6.99	27.05	32.81	36.68	41.07	48.52
children	39.36 ± 5.59	38.93 ± 5.90	39.14 ± 5.74	29.55	35.30	39.15	42.65	48.69
total sample	38.48 ± 6.63	37.84 ± 6.31	38.15 ± 6.47	27.73	34.11	37.88	41.79	48.65
Saturated fatty acids (%TE)								
toddlers	13.15 ± 3.71	13.57 ± 3.52	13.37 ± 3.62	7.69	11.26	13.10	15.59	19.19
children	14.04 ± 2.81	13.77 ± 3.09	13.90 ± 2.96	9.42	12.04	13.92	15.88	18.50
total sample	13.59 ± 3.32	13.67 ± 3.31	13.63 ± 3.31	8.36	11.59	13.60	15.73	18.96
Monounsaturated fatty acids (%TE)								
toddlers	11.47 ± 3.74	11.68 ± 2.80	11.58 ± 3.29	6.97	9.75	11.25	12.98	16.40
children	12.61 ± 2.58	12.64 ± 2.51	12.63 ± 2.53	8.82	11.01	12.44	14.11	17.02
total sample	12.04 ± 3.25	12.16 ± 2.70	12.10 ± 2.98	7.79	10.31	11.84	13.48	16.81
Polyunsaturated fatty acids (TE%)								
toddlers	7.56 ± 2.32	7.76 ± 2.67	7.66 ± 2.50	3.97	5.86	7.57	9.05	11.84
children	8.56 ± 2.60	8.37 ± 2.38	8.46 ± 2.49	4.55	6.81	8.46	9.94	12.53
total sample	8.06 ± 2.51	8.06 ± 2.55	8.06 ± 2.53	4.14	6.32	7.98	9.61	12.37
Dietary fiber (g)								
toddlers	11.47 ± 4.90	11.97 ± 4.87	11.73 ± 4.88	5.65	8.84	10.68	13.76	20.16
children	14.23 ± 5.43	14.22 ± 5.44	14.23 ± 5.42	7.23	10.57	13.38	17.11	23.91
total sample	12.84 ± 5.34	13.09 ± 5.27	12.97 ± 5.30	6.48	9.38	12.09	15.74	22.17

TE—total energy, P—percentile.

**Table 5:** Distribution of daily energy and macronutrient intake across geographical areas in a nationally representative sample of children 1–9 years of age living in Serbia (*n* = 576).

Variable Median (Range)	Belgrade (Capital City) Region	Vojvodina Region	Šumadija and Western Serbia Region	Southeastern Serbia Region
Energy (kcal)	1418.77 (594.89–2365.11)	1391.46 (648.82–2929.27)	1322.39 (595.27–2966.27)	1608.46 (738.51–3156.36) ***
Carbohydrates (%TE)	48.30 (32.08–67.91)	45.93 (14.84–75.60) **	47.78 (26.11–65.01)	48.07 (33.42–63.04)
Protein (%TE)	13.92 (8.26–21.51)	14.46 (7.09–23.24)	13.82 (8.35–22.86)	13.94 (9.18–19.13)
Fat (%TE)	37.20 (20.70–56.48)	38.46 (17.30–68.11)	37.79 (21.59–58.65)	37.41 (25.89–49.71)
Saturated fatty acids (%TE)	13.15 (3.46–20.95)	13.92 (3.49–24.88)	13.67 (5.20–28.27)	13.41 (8.69–24.56)
Monounsaturated fatty acids (%TE)	11.43 (4.27–22.24)	12.48 (5.48–29.71) **	11.73 (4.92–20.49)	11.73 (7.13–19.89)
Polyunsaturated fatty acids (TE%)	7.79 (1.83–15.49)	7.90 (2.59–16.19)	7.66 (3.18–21.52)	7.65 (2.71–15.87)
Dietary fiber (g)	11.38 (4.05–30.70)	11.53 (2.20–44.71)	10.78 (1.10–26.52)	13.72 (4.75–38.56) ***

** *p* < 0.01, *** *p* < 0.001, The Kruskal-Wallis H test; TE—total energy.

**Table 6:** Contribution of food groups to total energy intake and energy intake from carbohydrates, protein, fat, dietary fiber and saturated fatty acids across age and gender categories in a nationally representative sample of 1–3-year-old toddlers (*n* = 290) and 3–9-year-old children (*n* = 286) living in Serbia.

Food Groups $\left(\bar{x}\;\pm \;\mathrm{SD}\right)$	%TE	Carbohydrates (%TE)	Protein (%TE)	Fat (%TE)	Dietary Fibre (%TE)	Saturated FA (%TE)
Milk/milk products						
Toddlers	18.70 (13.77–24.56)	10.28 (6.15–15.13)	28.59 ± 11.84	26.63 (20.92–36.57)	0.04 ± 0.10	45.48 ± 15.64
Children	15.38 (10.62–19.66) ***	6.93 (4.48–11.09) ***	22.86 ± 9.38 ***	21.73 (14.96–29.48) ***	0.02 ± 0.06 *	38.46 ± 15.18 ***
Total sample	16.94 (12.21–21.86)	8.49 (4.96–12.97)	25.75 ± 11.06	24.33 (17.49–31.99)	0.03 ± 0.08	42.00 ± 15.79
Eggs/egg products						
Toddlers	3.07 (0.55–5.13)	0.14 (0.02–0.21)	7.60 (1.46–11.71)	5.14 (1.00–8.70)	0.01 ± 0.02	3.76 (0.73–6.93)
Children	2.86 (0.91–5.03)	0.13 (0.04–0.22)	6.84 (2.40–11.76)	4.47 (1.63–8.64)	0.01 ± 0.01	3.33 (1.19–6.42)
Total sample	3.00 (0.73–5.08)	0.13 (0.04–0.21)	7.22 (2.01–11.69)	4.86 (1.24–8.62)	0.01 ± 0.01	3.59 (0.87–6.64)
Meat/meat products						
Toddlers	9.46 (5.39–13.58)	0.05 (0.00–0.18)	24.13 ± 13.04	15.76 (8.70–24.29)	0.06 ± 0.10	14.87 (7.27–24.63)
Children	11.28 (7.14–15.81) ***	0.11 (0.01–0.24) ***	28.76 ± 12.58 ***	18.68 (10.22–26.75) **	0.08 ± 0.13 *	18.26 (10.09–28.25) **
Total sample	10.27 (6.12–14.93)	0.09 (0.00–0.22)	26.43 ± 13.01	17.08 (9.27–25.36)	0.07 ± 0.12	16.76 (8.49–26.45)
Seafood and related products						
Toddlers	1.30 ± 3.00	0.18 ± 0.54	3.11 ± 7.40	1.80 ± 4.62	0.01 ± 0.04	0.27 ± 0.69
Children	1.13 ± 2.80	0.23 ± 0.99 *	2.72 ± 6.55	1.40 ± 3.52	0.01 ± 0.03	0.23 ± 0.66
Total sample	1.23 ± 2.90	0.21 ± 0.80	2.92 ± 7.05	1.50 ± 4.32	0.01 ± 0.04	0.25 ± 0.67
Fat/oil						
Toddlers	9.22 ± 4.64	0.00 (0.00–0.01)	0.01 (0.00–0.04)	25.35 ± 11.55	─	12.32 (7.88–17.92)
Children	11.15 ± 4.89 ***	0.00 (0.00–0.02)	0.02 (0.00–0.08)	29.01 ± 11.64 ***	─	13.99 (8.75–21.30) *
Total sample	10.18 ± 4.86	0.00 (0.00–0.01)	0.01 (0.00–0.05)	27.17 ± 11.73	─	12.97 (8.51–19.93)
Grains/grain products						
Toddlers	30.27 ± 9.15	46.80 ± 12.23	23.08 ± 8.42	10.47 (5.59–16.39)	39.31 ± 14.50	6.54 (3.37–12.23)
Children	30.82 ± 8.84	49.81 ± 11.79 **	23.23 ± 7.86	8.48 (4.74–14.28) *	43.96 ± 15.45 ***	5.47 (2.27–9.71)*
Total sample	30.55 ± 8.99	48.29 ± 12.09	23.15 ± 8.14	9.60 (5.13–15.70)	41.62 ± 15.14	5.95 (2.82–10.58)
Nuts/seeds/kernel products						
Toddlers	0.04 (0.00–0.29)	0.05 (0.00–0.19)	0.03 (0.00–0.26)	0.01 (0.00–0.14)	0.21 (0.00–1.61)	0.02 (0.00–0.20)
Children	0.09 (0.02–0.77)	0.10 (0.03–0.35) ***	0.07 (0.02–0.55) ***	0.03 (0.01–0.53) *	0.62 (0.08–2.46) ***	0.03 (0.01–0.47) **
Total sample	0.05 (0.01–0.59)	0.07 (0.01–0.26)	0.05 (0.01–0.45)	0.02 (0.00–0.40)	0.42 (0.02–2.14)	0.02 (0.00–0.33)
Vegetables/vegetable products						
Toddlers	4.70 (3.06–6.94)	7.22 (4.26–10.86)	5.93 (3.58–9.17)	0.80 (0.50–1.25)	20.95 (13.63–31.24)	0.32 (0.17–0.55)
Children	5.11 (3.62–7.52)	8.31 (5.73–12.24) **	5.76 (3.33–8.95)	0.85 (0.52–1.31)	23.84 (16.00–33.79)	0.36 (0.20–0.58)
Total sample	4.91 (3.21–7.21)	7.96 (4.94–11.72)	5.80 (3.44–9.01)	0.81 (0.51–1.28)	22.31 (14.61–32.78)	0.34 (0.18–0.56)
Fruits/fruit products						
Toddlers	11.41 (6.94–14.80)	21.06 ± 11.29	2.93 (1.69–4.28)	1.08 (0.68–1.77)	31.65 ± 15.02	0.70 (0.36–1.13)
Children	7.90 (4.52–12.18) ***	16.07 ± 10.60 ***	2.07 (1.08–3.64) ***	0.67 (0.33–1.22) ***	23.52 ± 15.00 ***	0.41 (0.14–0.85) ***
Total sample	9.50 (5.35–13.86)	18.58 ± 11.22	2.59 (1.36–3.96)	0.88 (0.45–1.57)	27.61 ± 15.54	0.59 (0.26–1.02)
Sugar/sugar products						
Toddlers	2.69 (0.15–6.96)	4.80 (0.28–9.68)	0.12 (0.00–2.04)	0.47 (0.00–6.00)	0.00 (0.00–1.78)	0.74 (0.00–8.09)
Children	5.40 (2.22–9.81) ***	7.49 (3.45–13.17) ***	1.27 (0.05–3.20) ***	3.75 (0.00–9.47) ***	0.75 (0.00–2.59) ***	4.52 (0.00–13.42) ***
Total sample	4.31 (1.09–8.53)	6.09 (2.10–11.69)	0.67 (0.00–2.78)	2.10 (0.00–7.79)	0.23 (0.00–2.42)	2.89 (0.00–10.56)
Beverages (non–milk)						
Toddlers	1.50 ± 3.20	2.91 ± 5.62	0.08 ± 0.44	0.06 ± 0/36	0.01 ± 0.02	0.02 ± 0.20
Children	1.89 ± 2.92	3.88 ± 5.74	0.08 ± 0.31 *	0.05 ± 0.20	0.01 ± 0.01	0.01 ± 0.08
Total sample	1.75 ± 3.15	3.33 ± 5.74	0.08 ± 0.38	0.06 ± 0.29	0.01 ± 0.01	0.02 ± 0.16
Miscellaneous food products						
Toddlers	0.02 (0.00–0.13)	0.03 (0.00–0.19)	0.00 (0.00–0.33)	0.00 (0.00–0.01)	0.00 (0.00–0.38)	0.00 (0.00–0.00)
Children	0.16 (0.03–4.31) ***	0.26 (0.04–4.72) ***	0.40 (0.00–2.67) ***	0.03 (0.00–2.43) ***	0.28 (0.00–3.91) ***	0.00 (0.00–1.66) ***
Total sample	0.06 (0.00–1.25)	0.08 (0.00–1.71)	0.12 (0.00–1.23)	0.00 (0.00–0.67)	0.00 (0.00–0.95)	0.00 (0.00–0.46)
Products for special nutritional use						
Toddlers	1.90 ± 7.50	2.04 ± 7.74	1.73 ± 7.72	1.70 ± 7.47	0.09 ± 0.36	0.55 ± 2.47
Children	0.16 ± 1.76 ***	0.20 ± 2.34	0.04 ± 0.53 **	0.12 ± 1.57 **	0.01 ± 0.19	0.06 ± 0.92 ***
Total sample	1.03 ± 5.50	1.12 ± 5.84	0.89 ± 5.57	0.96 ± 5.41	0.04 ± 0.29	0.30 ± 1.88

TE—total energy intake; FA—fatty acids. Data are presented as average ± standard deviation for normally distributed data, and as median (range) for skewed data; differences between toddler and children groups were tested with Student’s *t*-test for normally distributed data and Mann–Whitney test in case of skewed distribution; * *p* < 0.05, ** *p* < 0.01, *** *p* < 0.001.

## Data Availability

Results attained in this study are included in the manuscript. Individual data are not available due to official legal, organizational and data security policies and ethical restrictions.
